# Penalized Maximum Likelihood Angular Super-Resolution Method for Scanning Radar Forward-Looking Imaging

**DOI:** 10.3390/s18030912

**Published:** 2018-03-19

**Authors:** Ke Tan, Wenchao Li, Qian Zhang, Yulin Huang, Junjie Wu, Jianyu Yang

**Affiliations:** School of Electronic Engineering, University of Electronic Science and Technology of China, No. 2006, Xiyuan Ave, West Hi-Tech Zone, Chengdu 611731, China; liwenchao@uestc.edu.cn (W.L.); cqzhangq92@163.com (Q.Z.); yulinhuang@uestc.edu.cn (Y.H.); junjie_wu@uestc.edu.cn (J.W.); jyyang@uestc.edu.cn (J.Y.)

**Keywords:** scanning radar forward-looking imaging, deconvolution, angular super-resolution, penalized maximum likelihood

## Abstract

Deconvolution provides an efficient technology to implement angular super-resolution for scanning radar forward-looking imaging. However, deconvolution is an ill-posed problem, of which the solution is not only sensitive to noise, but also would be easily deteriorate by the noise amplification when excessive iterations are conducted. In this paper, a penalized maximum likelihood angular super-resolution method is proposed to tackle these problems. Firstly, a new likelihood function is deduced by separately considering the noise in I and Q channels to enhance the accuracy of the noise modeling for radar imaging system. Afterwards, to conquer the noise amplification and maintain the resolving ability of the proposed method, a joint square-Laplace penalty is particularly formulated by making use of the outlier sensitivity property of square constraint as well as the sparse expression ability of Laplace distribution. Finally, in order to facilitate the engineering application of the proposed method, an accelerated iterative solution strategy is adopted to solve the obtained convex optimal problem. Experiments based on both synthetic data and real data demonstrate the effectiveness and superior performance of the proposed method.

## 1. Introduction

Forward-looking radar imaging is highly desirable in many applications, such as airplane navigation, automatic landing and material airdrop, etc. [1,2]. However, conventional monostatic synthetic aperture radar (SAR) and Doppler beam sharpen (DBS) techniques cannot be used for forwarding-looking imaging because it is difficult for them to acquire a high azimuth resolution [3,4]. The reason is that the gradient of the Doppler frequency in the forward-looking region is small, and the targets situated symmetrically about the flight path have the same Doppler history [5].

In order to obtain high-resolution image in forward-looking terrain, bistatic SAR whose transmitter and receiver are mounted on separate platform can be adopted [6,7]. However, bistatic SAR is confronted with complicated processing problems, such as synchronization and motion compensation [8,9]. Another efficient approach for forward-looking imaging is to use scanning radar to acquire the real beam image firstly. Then, the super-resolution techniques are conducted to improve the azimuth resolution [10]. In [11,12], the real iterative adaptive approach (Real-IAA) was introduced to the scanning radar to realize the super-resolution imaging, and it can greatly improve the angular resolution without introducing the false targets. However, since there are multiple matrix-vector multiplications and inverse matrix calculations, a great quantity of computation time is required, and this greatly restricts the application of Real-IAA.

Deconvolution is regarded as another simple and efficient technique to facilitate the super-resolution imaging for scanning radar [13,14]. Nevertheless, a multitude of deconvolution methods are sensitive to noise due to the inherent ill property. For example, the digital inverse filtering is used to improve the signal resolution, but it failed to dispose in the presence of noise [15]. An optimum finite impulse response (FIR) filter is developed in [16], which could improve azimuth resolution 23 times depending on high signal-to-noise ratio (SNR). In [17,18,19], a scheme of the truncated singular value decomposition (TSVD) method was proposed for radar forward-looking imaging and can significantly suppress the noise, whereas, the improvement for the resolution is limited and the number of reserved truncated values is difficult to determine.

The maximum likelihood (ML) methods provide effective approaches to ease the noise sensitivity problem by absorbing the statistical property of the imaging process [20,21]. In the ML methods, two noise processes are generally to be considered. The first models the data as a Gaussian process, such as the Landweber algorithm, which is also called the image space reconstruction algorithm (ISRA) in the astronomical area [22,23]. This method obtains a wide range of application for the universality of Gaussian noise. Another popular method is the Richardson–Lucy (RL) algorithm, which models the data as the Poisson process and is usually adopted by the astronomy, medical imaging, etc. [24,25]. Under such situations, the intensity of the received data is determined by the number of photons, and the Poisson distribution is therefore more suitable for the data modeling. However, these models only consider the amplitude of the echo and do not exactly match with the radar imaging system for ignoring the phase.

Moreover, in recent years, quite a few researchers have used the maximum a posteriori (MAP) framework to realize forward-looking super-resolution imaging [26,27]. These methods are conducted to absorb the additional prior information about the targets to obtain a higher resolution. In addition, the super-resolution performances of these methods did overmatch the traditional ML methods in terms of the resolving ability. Nevertheless, since the considered noise model is still based on the amplitude of the echo, the resulting imagery is degraded due to the appearance of false targets. In [28], the Doppler phase information is taken into account in the echo model and the sparse prior is introduced to enhance the resolve ability. However, the normal Gaussian distribution is adopted for the consideration of the noise process. Therefore, the spurious targets would still cause the results to deteriorate when it comes to the continuous scene.

Furthermore, oweing to the ill-posed nature of the deconvolution problem, the solutions of these algorithms exhibit large instabilities due to the noise amplification phenomenon and the false targets increasing when excessive iterations are conducted [29]. One way to overcome this difficulty is to interrupt the iterations before the instabilities appear by adopting an appropriate iterative stopping criterion, for instance, the discrepancy principle [30] and the relative improvement norm criterion [31], etc. However, in most of these criteria, a stopping threshold is normally required and it cannot always be determined accurately in practical applications, such as the noise norm for the discrepancy principle or the convergence tolerance for the relative improvement norm criterion. As a result, this inaccuracy may cause great performance deterioration if the super-resolution methods are sensitive to the iterations, such as the Landweber, RL and sparse-based MAP methods.

In this paper, a maximum likelihood method associated with a specially formulated penalty function is proposed to tackle these problems. Firstly, we establish the likelihood function by separately considering the noise in the I and Q channels to improve the accuracy of the noise modeling for a radar imaging system. Therefore, the resistance to the noise can be highly improved according to this likelihood function. Next, to conquer the noise amplification and enhance the resolving ability, a joint square-Laplacian-penalty is particularly developed by making use of the outlier sensitivity property of a square constraint as well as the sparse expression ability of Laplacian distribution. Finally, an accelerated iterative solution strategy is adopted to solve the obtained convex optimal problem to facilitate the engineering application for its advantage of simple implementation and fast convergence. Experiments based on both synthetic data and real data demonstrate that our proposed method can efficiently reduce the spurious targets introduced by the noise and enhance the robustness of the iterations to the noise amplification.

The rest of this paper is organized as follows: In Section 2, the signal model of forward-looking scanning radar is established. In Section 3, the likelihood is firstly deduced by separating considering the noise in I and Q channels. Then, the joint square-Laplacian-penalty is particularly formulated. Finally, an accelerated iterative solution strategy is adopted to solve the obtained convex optimal problem. The results of simulations and real measured data are given to demonstrate the advantage of the proposed method in Section 4. Section 5 concludes this paper.

## 2. Signal Model for Forward-Looking Scanning Radar

**Figure 1:**
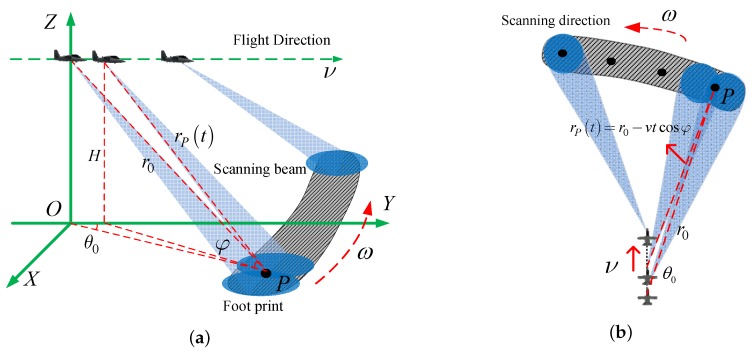
The geometry illustration of forward-looking scanning radar (not to scale). (**a**) oblique view (**b**) top view.

The airplane is flying along the *y*-axis at the speed of *v*, as shown in Figure 1. The radar scans its antenna in azimuth with rotational rate ω. Linear frequency modulation signals are transmitted and received at a certain pulse repetition frequency. After range pulse compression, the echo of target *P* can be expressed as a two dimensional complex signal

(1) $$s_{P}\left(\tau ,t\right)=\sigma_{0}h\left(t-t_{0}\right)\sin c\left[\tau -\frac{2r_{P}\left(t\right)}{c}\right]\exp\left\{-j\frac{4\pi }{\lambda }r_{P}\left(t\right)\right\},$$

where τ and *t* denote the range time and azimuth time, respectively, $\sigma_{0}$ denotes the scattering coefficient of target *P*, $t_{0}$ denotes the time when beam center scans *P*, *c* is the speed of light, λ is the wavelength of the transmitted electromagnetic wave, $h\left(t\right)$ represents the antenna pattern, and $r_{P}\left(t\right)$ represents the slant range history of target *P*.

As Figure 1 shows, the slant range history can be expressed as

(2) $$r_{P}\left(t\right)=\sqrt{r_{0}^{2}+{\left(vt\right)}^{2}-2r_{0}vt\cos\theta_{0}\cos\varphi },$$

where $r_{0}$ and $\theta_{0}$ are the initial slant range and azimuth angle of target *P*, and $\varphi_{0}$ is the incident angle. In practical applications, due to the narrow azimuth beam and fast scanning speed small imaging region, the slant range can be approximated as [32]

(3) $$r_{P}\left(t\right)\approx r_{0}-vt\cos\theta_{0}\cos\varphi .$$

Since the imaging sector is narrow and the azimuth angle for the scatters in the forward-looking area is less than $10^{\circ }$, we can approximate $\cos\theta_{0}$ as 1. Therefore, the range history can be simplified as

(4) $$r_{P}\left(t\right)\approx r_{0}-vt\cos\varphi .$$

In order to facilitate the analysis of spatial target echo, the time variables can be translated to the spatial variables based on $t=\left(\theta -\theta_{a}\right)/\omega$ and τ=2r/c. Combing Equation (4), Equation (1) can be converted to

(5) $$s_{P}\left(r,\theta \right)=\sigma_{0}h\left(\theta -\theta_{0}\right)\mathrm{sinc}\left\{\frac{2B}{c}\left[r-\left(r_{0}-v\frac{\theta -\theta_{a}}{\omega }\cos\varphi \right)\right]\right\}\exp\left\{-j\frac{4\pi }{\lambda }\left(r_{0}-v\frac{\theta -\theta_{a}}{\omega }\cos\varphi \right)\right\},$$

where $\theta_{a}$ denotes the initial scanning angle. For the area target with scattering function x(r,θ), the echo is the integral of all the point targets’ echo. Then, the echo of the area targets is

(6) $$s_{\Omega }\left(r,\theta \right)\;\;=\;\;\;\;\int \int \;\;\;\sigma (\bar{r},\bar{\theta })h\left(\theta \;-\;\bar{\theta }\right)\sin c\left\{\frac{2B}{c}\left[r\;-\;\left(\bar{r}\;-\;v\frac{\theta \;-\;\theta_{a}}{\omega }\cos\varphi \right)\;\right]\;\right\}\exp\;\left\{\;\;-j\frac{4\pi }{\lambda }\left(\;\bar{r}\;-\;v\frac{\theta \;-\;\theta_{a}}{\omega }\cos\varphi \right)\;\right\}d\bar{r}d\bar{\theta }.$$

In order to eliminate range migration, the linear transformation can be conducted for $r^{\prime }=r-\nu \frac{\theta -\theta_{a}}{\omega }$

(7) $${\tilde{s}}_{s}\left(r^{\prime },\theta \right)\;=\;\;\;\int \int \;\sigma (\bar{r},\bar{\theta })\exp\left\{-j\frac{4\pi }{\lambda }\bar{r}\right\}h\left(\theta -\bar{\theta }\right)\sin c\left\{\frac{2B}{c}\left(r^{\prime }-\bar{r}\right)\right\}d\bar{r}d\bar{\theta }\exp\left\{j\frac{4\pi }{\lambda }v\frac{\theta -\theta_{a}}{\omega }\cos\varphi \right\}.$$

Therefore, after approximation and range migration correction, the amplitude of the echo for the forward-looking scanning radar can be modeled as a two-dimensional detachable convolution of the scattering coefficients with the sinc function in the range direction and antenna pattern function in the azimuth direction. Since we concentrate on how to improve the azimuth resolution, only the azimuth signal is considered. If we extract the azimuth echo, a more concise expression can be obtained

(8) $$\begin{array}{cc} s_{s}\left(\theta \right) & =\;\;\;\;\int \;\sigma \left(\bar{\theta }\right)\;h\left(\theta -\bar{\theta }\right)\;\;d\bar{\theta }\exp\left\{j\frac{4\pi }{\lambda }\nu \frac{\theta -\theta_{a}}{\omega }\cos\varphi \right\}\\ & =\sigma \left(\theta \right)⊗h\left(\theta \right)\times \exp\left\{j\vartheta \left(\theta \right)\right\}, \end{array}$$

where ⊗ denotes the convolution operation, $\vartheta \left(\theta \right)=\frac{4\pi }{\lambda }\nu \frac{\theta -\theta_{a}}{\omega }\cos\varphi$ denotes the Doppler phase that only varies with the azimuth variable θ. Equation (8) illustrates that the angular resolution is coarsened due to the weighting effect of the antenna beam and is determined by

(9) $$\theta_{3\;\mathrm{dB}}\propto \lambda /D,$$

where $\theta_{3\;\mathrm{dB}}$ is the 3 dB width of antenna beam, and *D* is the antenna aperture size. Although the angular resolution for the scanning radar could be improved by increasing the physical radar antenna aperture, this method is constrained by the antenna weight, size, and other physical factors in many cases. Hence, the angular resolution is usually much worse than a typical range resolution. To improve the angular resolution, the deconvolution techniques can be adopted to obtain the super-resolution image with angular resolution in excess of the inherent resolution defined by the antenna beam width. In principle, the targets can be obtained by conducting the inverse filter on the amplitude of the azimuth echo

(10) $$\sigma \left(\varpi \right)=s\left(\varpi \right)/h\left(\varpi \right),$$

where $\sigma \left(\varpi \right)$, $s\left(\varpi \right)$ and $h\left(\varpi \right)$ are the Fourier transforms of σ(θ), s(θ) and h(θ), respectively, and ϖ is the angular spatial frequency. However, since $h\left(\varpi \right)$ is zero for the frequencies above some cutoff value, $1/h\left(\varpi \right)$ will be *∞* at those frequencies. In practice, it will result in tremendous amplification of noise and the inverse filter would become invalid due to this band limited character of $h\left(\varpi \right)$. In order to solve the ill-posed problem of deconvolution, the statistical modeling method is normally adopted.

## 3. Methodology

The principle of the statistical modeling method for deconvolution is to treat all the targets and observed data as stochastic unknown variables and the joint probability distribution is defined by incorporating the statistical information of these variables. Therefore, we formulate the deconvolution problem as maximizing an objection function depending on the likelihood, which measures the fitness of the estimation with the observed data and a constraint allowing us to bias the solution to a particular solution

(11) $$\hat{\sigma }=\underset{\sigma }{argmax}F\left(\sigma \right),$$

where $F\left(\sigma \right)$ is the objective function following the form

(12) $$F\left(\sigma \right)\;=F_{1}\left(\sigma \right)\;+\eta F_{2}\left(\sigma \right).\;$$

The term $F_{1}\left(\sigma \right)$ denotes the likelihood function that is determined by particular noise process, while $F_{2}\left(\sigma \right)$ is the penalty term, which is utilized to incorporate the prior information about the estimator to constrain the solution. The parameter η controls the relative weight of the two terms.

In this section, we will firstly derive the likelihood function according to the data acquisition process by separately considering the noise in I and Q channels. Afterwards, a joint square-Laplacian-penalty term is developed to improve the resolving ability of the algorithm and enhance its robustness to the noise amplification. Finally, to facilitate the engineering application of proposed algorithm, an accelerated iterative shrinkage/thresholding solution strategy is adopted to solve the obtained optimal problem.

### 3.1. Likelihood

The likelihood describes the process of data acquisition and is determined by the probability of obtaining the echo on the premise of given targets. Therefore, in the framework of maximum likelihood theory, the statistical properties of the measured data are mainly influenced by the noise. In radar imaging, the system noise is produced by the thermal motion of electrons and normally obeys the Gaussian distribution. After demodulation, the system noise is fed into I and Q channels, respectively, which are uncorrected to each other and still obey the Gaussian distribution. Hence, considering the effect of noise, the azimuth echo can be rewritten as

(13) $$\tilde{s}\left(\theta \right)=\left(\sigma \left(\theta \right)⊗h\left(\theta \right)\cdot \cos\left\{\vartheta \left(\theta \right)\right\}+n_{c}\left(\theta \right)\right)+j\left(\sigma \left(\theta \right)⊗h\left(\theta \right)\cdot \sin\left\{\vartheta \left(\theta \right)\right\}+n_{s}\left(\theta \right)\right),$$

where $n_{c}\left(\theta \right)$ and $n_{s}\left(\theta \right)$ are the noise existed in I/Q channels, respectively. For mathematical analysis, the continuous convolution model could be discretized to a matrix vector form as follows:

(14) $$\tilde{s}=\left(H\sigma ⊙\cos\left(\vartheta \right)+n_{c}\right)+j\left(H\sigma ⊙\sin\left(\vartheta \right)+n_{s}\right),$$

where $s={\left[s_{1},\ldots ,s_{N}\right]}^{T}$ represents the received data in one range cell, *N* is the length of the vector, $\sigma ={\left[\sigma_{1},\ldots ,\sigma_{N}\right]}^{T}$ represents the scattering coefficient to be estimated, $\vartheta ={\left[\vartheta_{1},\ldots ,\vartheta_{N}\right]}^{T}$ represents the Doppler phase, $n={\left[n_{1},\ldots ,n_{N}\right]}^{T}$ denotes the noise, and H denotes a convolution operation matrix that derives from the azimuth antenna pattern $h={\left[h_{1},\ldots ,h_{L}\right]}^{T}$

(15) $$H=\left[\begin{array}{ccccccc} h_{1} & & & & h_{L} & \cdots & h_{2}\\ h_{2} & h_{1} & & & & \ddots & h_{3}\\ \vdots & & \ddots & & & & \vdots \\ h_{L} & & & & & & \\ & \ddots & & & & & \\ & & \ddots & & & & \\ & & & h_{L} & \cdots & h_{2} & h_{1} \end{array}\right].$$

We take an arbitrary sample point to derive the probability distribution of the random amplitude. For the given pixel *i*, the statistical property of the received data ${\tilde{s}}_{i}$ can be derived by separately considering the noise in the I and Q channels

(16) $${\tilde{s}}_{i}=\left({\left(H\sigma \right)}_{i}\cos\left(\vartheta_{i}\right)+n_{c_{i}}\right)+j\left({\left(H\sigma \right)}_{i}\sin\left(\vartheta_{i}\right)+n_{s_{i}}\right).$$

Rewrite Equation (16) in complex signal

(17) $${\tilde{s}}_{i}=s_{i}\exp\left\{j\phi_{i}\right\},$$

where $s_{i}$ and $\phi_{i}$ are the random amplitude and random phase, respectively. It can be observed from Equation (17) that $s_{i}$ and $\phi_{i}$ are the two-dimensional transformation of $n_{c_{i}}$ and $n_{s_{i}}$

(18) $$\left\{\begin{array}{c} s_{i}\cos\left(\phi_{i}\right)={\left(H\sigma \right)}_{i}\cos\left(\vartheta_{i}\right)+n_{c_{i}},\\ s_{i}\sin\left(\phi_{i}\right)={\left(H\sigma \right)}_{i}\sin\left(\vartheta_{i}\right)+n_{s_{i}}. \end{array}\right.$$

Therefore, to derive the PDF of $s_{i}$, the Jacobian determinant determined by the transformation should be firstly calculated

(19) $$\left|J\right|=\left|\begin{array}{cc} \frac{\partial n_{c_{i}}}{\partial s_{i}} & \frac{\partial n_{c_{i}}}{\partial \phi_{i}}\\ \frac{\partial n_{s_{i}}}{\partial s_{i}} & \frac{\partial n_{s_{i}}}{\partial \phi_{i}} \end{array}\right|=\left|\begin{array}{cc} \cos\left(\phi_{i}\right) & -s_{i}\sin\left(\phi_{i}\right)\\ \sin\left(\phi_{i}\right) & s_{i}\cos\left(\phi_{i}\right) \end{array}\right|=s_{i}.$$

Therefore, we can get the joint PDF of $s_{i}$ and $\phi_{i}$ following the transformation formula of two-dimensional random variables

(20) $$f_{S_{i}\Phi_{i}}\left(s_{i},\phi_{i}\right)=\left|J\right|f_{N_{c_{i}}N_{s_{i}}}\left(n_{c_{i}},n_{s_{i}}\right).$$

Since the Gaussian random variables $n_{c_{i}}$ and $n_{s_{i}}$ are uncorrelated to each other, the joint PDF of $n_{c_{i}}$ and $n_{s_{i}}$ can be determined by the simple multiplication of the PDF of $n_{c_{i}}$ and $n_{s_{i}}$, both of which obey the Gaussian distributions with zero-mean and standard deviation ρ. Therefore,

(21) $$f_{S_{i}\Phi_{i}}\left(s_{i},\phi_{i}\right)=s_{i}\cdot \frac{1}{\sqrt{2\pi }\rho }e^{-\frac{{\left(n_{c_{i}}\right)}^{2}}{2\rho^{2}}}\frac{1}{\sqrt{2\pi }\rho }e^{-\frac{{\left(n_{s_{i}}\right)}^{2}}{2\rho^{2}}}.$$

Taking the two-dimensional transformation of $n_{c_{i}}$ and $n_{s_{i}}$ in Equation (18) into Equation (21), we have

(22) $$\begin{array}{c} f_{S_{i}\Phi_{i}}\left(s_{i},\phi_{i}\right)=\frac{s_{i}}{2\pi \rho^{2}}e^{-\frac{{\left(s_{i}\cos\left(\phi_{i}\right)-{\left(H\sigma \right)}_{i}\cos\left(\vartheta_{i}\right)\right)}^{2}+{\left(s_{i}\sin\left(\phi_{i}\right)-{\left(H\sigma \right)}_{i}\sin\left(\vartheta_{i}\right)\right)}^{2}}{2\rho^{2}}}\\ =\frac{s_{i}}{2\pi \rho^{2}}e^{-\frac{{\left(s_{i}\right)}^{2}+{\left({\left(H\sigma \right)}_{i}\right)}^{2}-2\cos\left(\phi_{i}-\vartheta_{i}\right)}{2\rho^{2}}}. \end{array}$$

Integration with respect $\phi_{i}$ yields the marginal probability density of $s_{i}$

(23) $$\begin{array}{c} f_{S_{i}}\left(s_{i}\right)=\int_{0}^{2\pi }f_{S_{i}\Phi_{i}}\left(s_{i},\phi_{i}\right)\;\;d\;\phi_{i}\\ =\int_{0}^{2\pi }\frac{s_{i}}{2\pi \rho^{2}}e^{-\frac{{\left(s_{i}\right)}^{2}+{\left({\left(H\sigma \right)}_{i}\right)}^{2}-2\cos\left(\phi_{i}-\vartheta_{i}\right)}{2\rho^{2}}}\;\;d\;\phi_{i}\\ =\frac{s_{i}}{2\pi \rho^{2}}e^{-\frac{{\left(s_{i}\right)}^{2}+{\left({\left(H\sigma \right)}_{i}\right)}^{2}}{2\rho^{2}}}\int_{0}^{2\pi }e^{\frac{2\cos\left(\phi_{i}-\vartheta_{i}\right)}{2\rho^{2}}}\;\;d\;\phi_{i}. \end{array}$$

By utilizing the technique of residue integration, the integration term of Equation (23) can be calculated [33]

(24) $$\int_{0}^{2\pi }e^{\frac{2\cos\left(\phi_{i}-\vartheta_{i}\right)}{2\rho^{2}}}\;\;d\;\phi_{i}=2\pi J_{0}\left(\frac{s_{i}{\left(H\sigma \right)}_{i}}{\rho^{2}}\right),$$

where $J_{0}\left(\cdot \right)$ is the zero order Bessel function. Then, the final results of Equation (23) are

(25) $$f_{S_{i}}\left(s_{i}\right)=\frac{s_{i}}{\rho^{2}}e^{-\frac{{\left(s_{i}\right)}^{2}+{\left({\left(H\sigma \right)}_{i}\right)}^{2}}{2\rho^{2}}}\cdot J_{0}\left(\frac{s_{i}{\left(H\sigma \right)}_{i}}{\rho^{2}}\right).$$

Considering that the random variable $s_{i}$ are independent from each other and obey the same distribution, the joint PDF of the sampled data can be obtained by multiplying the individual PDF

(26) $$f_{S_{i}}\left(s_{i}\right)=\frac{s_{i}}{\rho^{2}}e^{-\frac{{\left(s_{i}\right)}^{2}+{\left({\left(H\sigma \right)}_{i}\right)}^{2}}{2\rho^{2}}}\cdot J_{0}\left(\frac{s_{i}{\left(H\sigma \right)}_{i}}{\rho^{2}}\right).$$

Our objective is to find the most likely $\sigma =\left[\sigma_{1},\sigma_{2},\ldots ,\sigma_{N}\right]$ to yield the observed data $s=\left[s_{1},s_{2},\ldots ,s_{N}\right]$, which means to maximize the probability of obtaining the data. To simplify this maximum procedure, we take the logarithm of joint PDF and the objective function is obtained

(27) $${F_{1}}^{\prime }\left(\sigma \right)=\ln\left(\prod_{i=1}^{N}\frac{s_{i}}{\rho^{2}}e^{-\frac{{\left(s_{i}\right)}^{2}+{\left({\left(H\sigma \right)}_{i}\right)}^{2}}{2\rho^{2}}}\cdot J_{0}\left(\frac{s_{i}{\left(H\sigma \right)}_{i}}{\rho^{2}}\right)\right).$$

Expanding Equation (27) and discarding the term that is independent with σ, we obtain the final likelihood function

(28) $$F_{1}\left(\sigma \right)=\sum_{i=1}^{N}\ln J_{0}\left(\frac{s_{i}{\left(\sigma \right)}_{i}}{\rho^{2}}\right)-\sum_{i=1}^{N}\frac{{\left({\left(\sigma \right)}_{i}\right)}^{2}}{2\rho^{2}}.$$

### 3.2. Joint Square-Laplacian Penalty

In this subsection, we will discuss the constraint posed on the solution. Normally, different constraints can result in different solutions. For instance, the L2 norm penalty is normally used to stabilize the solution by constraining the energy of results [34]. However, the results obtained by the L2 norm penalty tend to be smoothed and thus cannot distinguish the detail of the scene. Recently, the sparse prior has drawn much attention in radar imaging area for its powerful ability in distinguishing the targets [35]. In [36,37,38], the reconstruction for dominate sparse targets has been widely researched in Tomographic SAR, SAR and Inverse SAR (ISAR) imaging and enhanced imaging results have been acquired. In the forward-looking imaging area, the spare Bayesian methods have exhibited extraordinary ability in resolving the targets and reducing the sidelobe of the scatters. In those methods, the sparse prior is described by a Laplace distribution

(29) $$P\left(\sigma \right)=\frac{1}{\sqrt{2}\gamma }\exp\left(-\frac{\sqrt{2}\left|\sigma \right|}{\gamma }\right),$$

where $\sigma_{i}$ is the *i*-th element of the target, and γ denotes the scale parameter. Since the Laplace function owns the heavy tail characteristics, the energy of the targets are encouraged to be distributed in the larger value positions. Therefore, the targets tends to concentrate its energy in the sparse positions and this can greatly facilitate the resolving ability of the algorithm. Nevertheless, the Laplace prior cannot suppress the outlier and the noise amplification phenomenon will become serious while excessive iterations are conducted. One approach to solve this problem is to terminate the iterations before the noise amplification gets severe and some iterative termination criteria have been adopted by the researchers—for instance, the discrepancy principle and the relative improvement norm criterion, etc. However, those methods depend on accurate determination of the iterative stopping values (ISV) and the fluctuation of the ISV will result in great variation in the super-resolution performance. Therefore, to tackle this problem, a joint square-Laplacian-penalty is proposed by making use of the outlier sensitivity property of square constraint. The prior term can be modified as

(30) $$P\left(\sigma \right)=\frac{1}{2\sqrt{\pi }\gamma_{1}\gamma_{2}}\exp\left(-\frac{\sqrt{2}\left|\sigma \right|}{\gamma_{1}}-\frac{\sigma^{2}}{\gamma_{2}^{2}}\right),$$

where $\gamma_{1}$ denotes the scale parameter of Laplace distribution, and $\gamma_{2}$ denotes the standard deviation of Gaussian distribution. Since the square penalty is sensitive to outlier, the false targets and structures can be suppressed when excessive iterations are conducted. Therefore, by imposing this joint penalty to the solution, the estimated targets cannot have a strong ability in resolving targets, but also be more robust to the noise. Therefore, we can get the penalty term of Equation (12) through taking the log of the joint PDF of $\sigma_{i}$:

(31) $${F_{2}}^{\prime }\left(\sigma \right)\;=\log\left(\prod_{i=1}^{N}P\left(\sigma_{i}\right)\right)=\log\left(\prod_{i=1}^{N}\frac{1}{2\sqrt{\pi }\gamma_{1}\gamma_{2}}\exp\left(-\frac{\sqrt{2}\left|\sigma_{i}\right|}{\gamma_{1}}-\frac{{\sigma_{i}}^{2}}{\gamma_{2}^{2}}\right)\right).$$

Expanding Equation (31) and discarding the terms that are independent with σ, we obtain the final penalty term

(32) $$F_{2}\left(\sigma \right)=-\eta_{1}\sum_{i=1}^{N}\left|\sigma_{i}\right|-\eta_{2}\sum_{i=1}^{N}\sigma_{i}^{2},$$

where $\eta_{1}=\frac{\sqrt{2}}{\gamma_{1}}$ and $\eta_{2}=\frac{1}{\gamma_{2}^{2}}$ are control of the relative weight of the two penalty terms, respectively. The role of $F_{2}\left(\sigma \right)$ play in the $F\left(\sigma \right)$ is similar to the function of the regularization items playing in the regularization methods. Therefore, the determination for the parameters $\eta_{1}$ and $\eta_{2}$ can refer to the methods adopted in the regularization method, such as the L-curve method [39]. In our framework, the $\eta_{1}$ can be firstly fixed by an experienced value and then determine the value of $\eta_{2}$ through the L-curve method. Then, fix $\eta_{2}$ by the obtained value and also modify the value of $\eta_{1}$ through the L-curve method.

### 3.3. Solution for the Optimal Problem

After the derivation for likelihood and the penalty term, we obtain the final objective function

(33) $$\hat{\sigma }=\underset{\sigma }{argmax}\;\left\{F\left(\sigma \right)=\sum_{i=1}^{N}\ln J_{0}\left(\frac{s_{i}{\left(H\sigma \right)}_{i}}{\rho^{2}}\right)-\sum_{i=1}^{N}\frac{{\left({\left(H\sigma \right)}_{i}\right)}^{2}}{2\rho^{2}}-\eta_{1}\sum_{i=1}^{N}\left|\sigma_{i}\right|-\eta_{2}\sum_{i=1}^{N}\sigma_{i}^{2}\right\}.$$

Through minimizing this newly formulated objective function, the obtained solution can not only have a strong ability to resist the impact of noise, but also be more robust to the noise amplification. As to the solution of this optimal problem, since there are nonlinear Bessel functions and absolute value terms in the objection function, the direct inversion method is infeasible. To approximate the true solution and facilitate the engineering application of the proposed method, the idea of an accelerated iterative shrinkage/thresholding solution strategy proposed by us is adopted to solve this convex optimal problem for its convergence efficiency [40]. To implement the strategy, the gradient of the objective function should be firstly calculated. Since the absolute value term is non-differential, it can be firstly modified by the following smooth approximation:

(34) $$\sum_{i=1}^{N}\left|\sigma_{i}\right|=\sum_{i=1}^{N}\sqrt{\left({\sigma_{i}}^{2}+\varepsilon \right)}a.$$

ε≥0 is a small constant introduced to solve the non-differentiability of the absolute value term. Then, the gradient of objective function can be calculated as

(35) $$\nabla F\left(\sigma \right)\;=\frac{1}{\rho^{2}}H^{T}\left[\frac{J_{1}\left(\frac{s_{i}{\left(H\sigma \right)}_{i}}{\rho^{2}}\right)}{J_{0}\left(\frac{s_{i}{\left(H\sigma \right)}_{i}}{\rho^{2}}\right)}⊙s\right]-\frac{1}{\rho^{2}}H^{T}H\sigma -\eta_{1}diag\left\{{\left({\left|{\left(\sigma_{k}\right)}_{i}\right|}^{2}+\varepsilon \right)}^{-\frac{1}{2}}\right\}\sigma_{k}-2\eta_{2}\sigma_{k}\;\;,$$

where $diag\left(\cdot \right)$ is the diagonal matrix with the elements in the bracket as its diagonal. Following the gradient direction in the N-dimensional data space, the basic iterative formula to search the optimal solution is given by

(36) $$\sigma_{k+1}\;=\;\sigma_{k}\;+\;\beta \left[\frac{1}{\rho^{2}}H^{T}\left(\frac{J_{1}\left(\frac{s_{i}{\left(H\sigma_{k}\right)}_{i}}{\rho^{2}}\right)}{J_{0}\left(\frac{s_{i}{\left(H\sigma_{k}\right)}_{i}}{\rho^{2}}\right)}⊙s\right)\;-\;\frac{1}{\rho^{2}}H^{T}H\sigma_{k}\;-\;\eta_{1}diag\left\{{\left({\left|{\left(\sigma_{k}\right)}_{i}\right|}^{2}+\varepsilon \right)}^{-\frac{1}{2}}\right\}\sigma_{k}\;-\;2\eta_{2}\sigma_{k}\;\right],$$

where $\sigma_{k}$ are the iterative results after *k* iterations. β is a constant step size, which is confined to be smaller than $2/∥H^{T}H∥$ to ensure the convergence. ${ℜ}_{\delta }$: $R^{N}\to R^{N}$ is the shrinkage-thresholding operator defined by ${ℜ}_{\delta }\left(\sigma \right)=\left[{ℜ}_{\delta }\left(\sigma_{1}\right),\cdots ,{ℜ}_{\delta }\left(\sigma_{i}\right),\cdots ,{ℜ}_{\delta }\left(\sigma_{N}\right)\right]$, and

(37) $${ℜ}_{\delta }\left(\sigma_{i}\right)=\left\{\begin{array}{c} 0\;\;\;\;\;\;\;\;\;\;\;\;\;\;\;\;\;\;\;\;\;\;\;\;\;\;\;\;\;\;\;\;\;\;\;\;\;\;\;\;\;\;\;\;\;\;\;\;\;\;\;\;\;\;\;\;\;\;\;\;\;\;\;\;\;\;,\;\;\;\;\;\;\left|\sigma_{i}\right|\leq \delta ,\\ \sigma_{i}-\delta \mathrm{sgn}\left(\sigma_{i}\right)\;\;\;\;\;\;\;\;,\;\;\;\;\;\;\mathrm{otherwise}. \end{array}\right.$$

Then, the implementation steps to obtain the optimal solution of Equation (11) are given in the Table 1.

Compared with the traditional statistical methods, the results obtained by the proposed penalized maximum likelihood (PML) method can not only have a strong ability in resisting the noise, but also be more powerful in conquering the noise amplification as well as maintaining a good resolving ability.

## 4. Numerical Results

In this section, we present both the simulation experiment and the real data processing to illustrate the advantage of the proposed PML angular super-resolution method for forward-looking scanning radar. We compare the proposed method with the Landweber, Richardson–Lucy (RL) and the sparse MAP method.

### 4.1. Simulation Experiment

**Figure 2:**
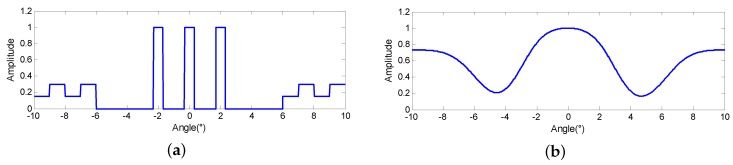
Simulation experiment (**a**) simulated scene; (**b**) echo.

Since we concentrate on improving the angular resolution, the simulated scene is set on the same range cell and is demonstrated in Figure 2a. It is composed of three extended targets located at $-2^{\circ }$, $0^{\circ }$ and $2^{\circ }$ and continuous scenes located in the background from $-10^{\circ }$ to $-6^{\circ }$ and $10^{\circ }$ to $6^{\circ }$, respectively. Some related parameters are demonstrated as Table 2. Figure 2b displays the amplitude of azimuth echo, from which it can be observed that the closely located targets cannot be distinguished and the outline of the continuous scenes are smoothed.

#### 4.1.1. Effectiveness Verification for Noise Resistance

**Figure 3:**
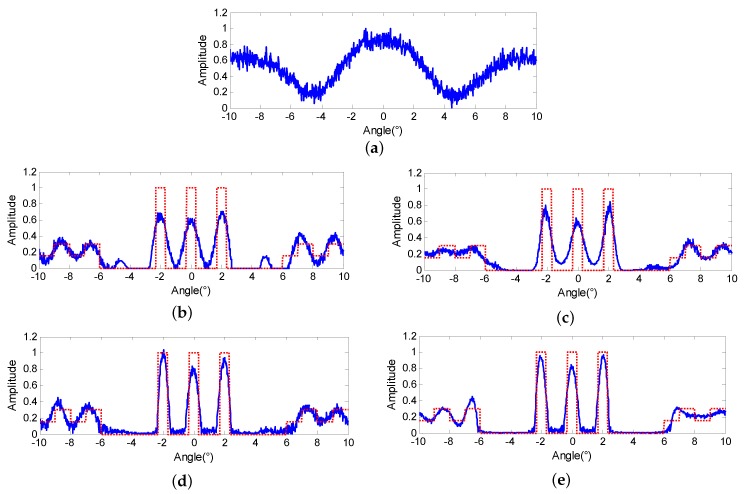
Super-resolution results comparison in the case of SNR = 20 dB (**a**) echo super-resolution results of (**b**) Landweber (**c**) Richardson-Lucy (RL) (**d**) maximum a posterior (MAP) (**e**) proposed penalized maximum likelihood (PML).

In this subsection, we aim to validate the advantage of the proposed method in terms of noise resistance. In order to properly fit the production process of the noise in radar imaging system, the noise are added to the echo through I and Q channels separately and the contaminated echo is shown in Figure 3a, of which the signal to noise ratio (SNR) equals 20 dB.

Four different statistical super-resolution methods are conducted on the echo of Figure 3a, and the discrepancy criterion is used to stop the iterations, in which the iteration number *k* is determined when the super-resolution results meet the following conditions:

(38) $${∥s-H\sigma_{k}∥}_{2}\leq \kappa ,$$

where κ is the iterative stopping value (ISV) and is normally determined by the estimate of the L2-norm of the noise [30]. We firstly compare the results from a visual perspective. Figure 3b,c are the results of the Landweber and RL, respectively, in which the blue line denotes the super-resolution results and the red dotted line represents the original scene. From the results, we can observe that both algorithms can separate the targets in the middle of the scene and retrieve the continuous background to some extent. However, the three extended targets cannot fit the original targets satisfactorily and the spurious targets emerge in Figure 3b. The reason is that both Landweber and RL are based on the maximum likelihood criterion and the noise modeling cannot match the radar system properly. Hence, the noise would deteriorate the super-resolution results when the ML criterion drives the results to approach the agreement with the contaminated data.

Figure 3d shows the results of the MAP method described in [26], and the regularization parameter is determined as 0.003 by the L-curve method. Compared with Figure 3b,c, the targets are separated more thoroughly for making use of the sparse prior information. However, since the noise model still cannot match the radar system exactly, the background is perplexed by the noise. The results of the proposed PML are demonstrated in Figure 3e and the regularization parameters are determined as 0.004 and 0.0015, respectively. It can be observed from Figure 3e that not only the targets can fit the original targets properly, but also the background is much clearer.

**Figure 4:**
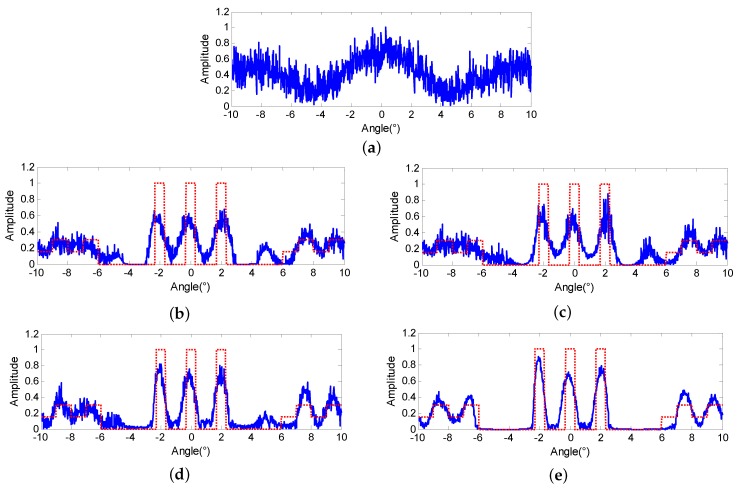
Super-resolution results comparison in the case of SNR = 10 dB (**a**) echo super-resolution results of (**b**) Landweber (**c**) Richardson-Lucy (RL) (**d**) maximum a posterior (MAP) (**e**) proposed penalized maximum likelihood (PML).

In order to verify the performance of the proposed method in the low SNR cases, stronger noise is added to the echo. The amplitude of the echo is shown in Figure 4a and the SNR equals 10 dB. Then, the four super-resolution methods are conducted on Figure 4a, respectively. Figure 4b,c, display the results of Landweber and RL, in which the results are seriously contaminated by the noise and more false targets appear. The results of MAP are displayed in Figure 4d and the regularization parameter is determined as 0.007. From Figure 4e, we can observe that the targets are separated more thoroughly. However, the background is more seriously perplexed by the spurious targets induced by the noise. Figure 4e demonstrates the results of the proposed PML and the regularization parameters are determined as 0.0085 and 0.0035, respectively. From Figure 4e, it can be observed that the results exhibit much clearer targets and the background and is pretty competitive compared with the results of Landweber, RL and MAP.

To further evaluate the performance of different super-resolution methods quantitatively, the relative error (ReErr) and structure similarity (SSIM) are used to measure the quality of the obtained results, which are defined as follows:

(39) $$\mathrm{ReErr}=\frac{{∥\hat{\sigma }-\sigma ∥}_{2}}{{∥\sigma ∥}_{2}},$$

(40) $$\mathrm{SSIM}=\frac{\alpha_{\hat{\sigma }\sigma }\mu_{\hat{\sigma }}\mu_{\sigma }}{\left(\mu_{\hat{\sigma }}^{2}+\mu_{\sigma }^{2}\right)\left(\alpha_{\hat{\sigma }}^{2}+\alpha_{\sigma }^{2}\right)},$$

where $\hat{\sigma }$ and σ correspond to the obtained super-resolution results and the original scene, respectively. The terms $\mu_{\hat{\sigma }}$, $\mu_{\sigma }$ and $\alpha_{\hat{\sigma }}$, $\alpha_{\sigma }$ denote the mean and standard deviation of $\hat{\sigma }$ and σ. $\alpha_{\hat{\sigma }\sigma }$ is the cross correlation of $\hat{\sigma }$ and σ after removing their means

(41) $$\alpha_{\hat{\sigma }\sigma }=\frac{1}{N}\sum_{i=1}^{N}\left({\hat{\sigma }}_{i}-\mu_{\hat{\sigma }}\right)\left(\sigma_{i}-\mu_{\sigma }\right).$$

ReErr measures the energy difference between the imaging results and the original scene. The smaller the value is, the better the results will be. SSIM measures the structure similarity between the results and the original scene. The larger the value is, the closer the results approach the original scene.

**Figure 5:**
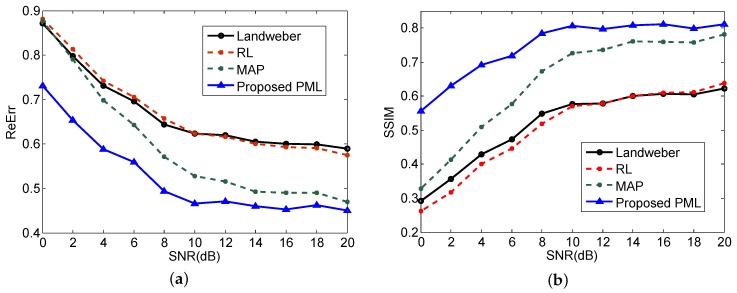
Quantitative comparison results of **(a**) relative error (ReErr); (**b**) structure similarity (SSIM).

The ReErr and SSIM obtained by the four super-resolution methods with different SNR are plotted in Figure 5a,b respectively. Only the results of relatively low SNR cases are showed for the purpose of illustrating the noise resistance advantage of the proposed method. Every point in the curves is obtained by the mean of 1000 Monte Carlo trials.

From Figure 5a, we can observe that the proposed PML method has the lowest ReErr in all SNR cases, which indicates that the super-resolution results of the proposed method are better than that of the other three methods in terms of precision. Furthermore, the ReErr of Landweber, RL and MAP are approaching a similar value when the SNR is decreasing to 0. It suggests that the super-resolution performance of these three methods would deteriorate to the same level in a low SNR situation. On the contrary, the proposed method behaves much better in the low SNR cases and the ReErr is much smaller than the other three methods. The SSIM results demonstrated in Figure 5b also verified the comparison results of ReErr. We can observe from Figure 5b that the proposed PML owns the largest SSIM in all SNR cases. Furthermore, when the SNR decreases, the SSIM of PML do not decline a lot. It suggests that our proposed method has a more prominent advantage in the low SNR situation and possesses a stronger ability to resist noise.

#### 4.1.2. Effectiveness Verification for Iterative Robustness

**Figure 6:**
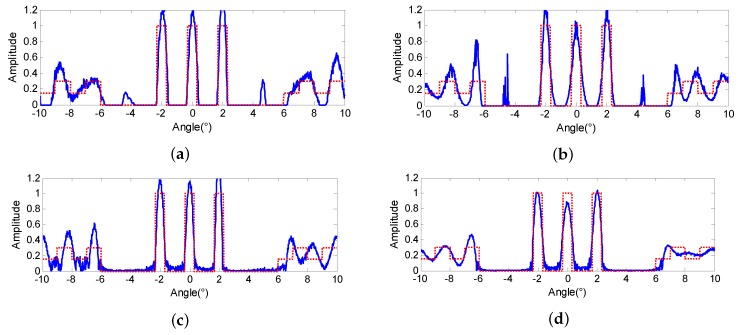
Super-resolution results of (**a**) Landweber (**b**) Richardson-Lucy (RL) (**c**) maximum a posterior (MAP) (**d**) proposed penalized maximum likelihood (PML) when the ISV equals 0.95κ with SNR = 20 dB.

In practical application, the κ cannot always be estimated exactly and the fluctuation of κ will result in super-resolution performance variation if the methods are sensitive to the number of iterations. To verify the advantage of the proposed method in terms of the robustness to the iterations, we modify the ISV to 0.95κ and conduct the four super-resolution methods once again on the echo of Figure 3a with the same regularization parameters, respectively. Figure 6b–d display the results of Landweber, RL and MAP when the iterations are terminated, in which the three extended targets have been overly retrieved and exceed the original values. In addition, there are some obvious false targets emerging and the outline of the continuous scene has been destroyed. However, the results of the proposed PML displayed in Figure 6d are quite similar to Figure 3e. The performance of PML presents itself to be much more stable when the ISV varies, and this would greatly facilitate its engineering application of the proposed method.

To demonstrate the variation of the super-resolution results versus the iterations deeply, the mean square errors (MSE) are calculated at every iteration

(42) $$\mathrm{MSE}=\sqrt{{\left(\sigma_{k}-\sigma \right)}^{2}/N}.$$

**Figure 7:**
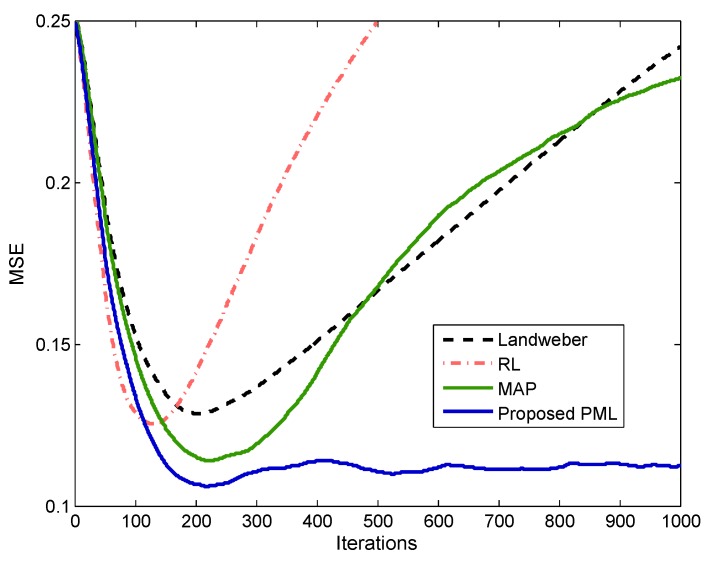
Mean square errors (MSE) curves comparison when SNR = 20 dB.

The MSE of the four methods are illustrated in Figure 7, respectively. It can be observed that the MAP has a smaller minimum MSE (MMSE) compared with Landweber and RL due to the introduction of the sparse prior. However, all the MSE curves of these three methods would diverge from the MMSE if too many iterations are performed. It suggests that the super-resolution performances would decline and distort from the optimal solution if the iterative cannot stop at a proper iteration. As a contrast, the proposed PML not only has the smallest minimum MSE, but also possesses a more stable MSE curve. Therefore, the proposed method can be more robust to resist the noise and give a more relaxed requirement for the iteration stopping criterion.

**Figure 8:**
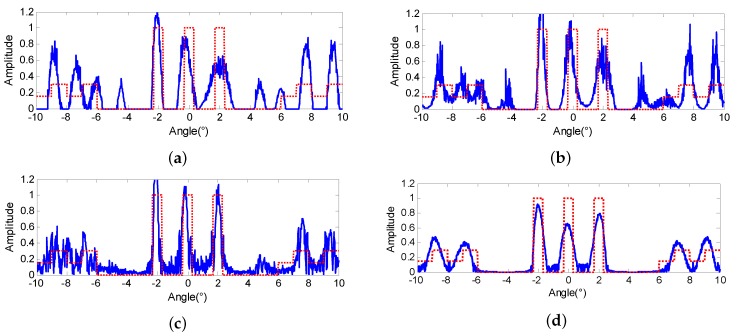
Super-resolution results of (**a**) Landweber (**b**) Richardson-Lucy (RL) (**c**) maximum a posterior (MAP) (**d**) proposed penalized maximum likelihood (PML), when the ISV equals 0.95κ with SNR = 10 dB.

Similar to the SNR=20dB cases, the iteration robustness of the proposed method is also investigated in the low SNR case. The four super-resolution methods are conducted once again on the echo of Figure 4a by modifying the ISV to 0.95κ. The results of Landweber, RL and MAP are displayed in Figure 8b–d, respectively, from which we can observe that the super-resolution results seriously deteriorate. Not only the three extended targets are distorted, but also the noise is amplified greatly. Many false targets emerge and some of them even surpass the original scene. However, the results of proposed PML are presented to be pretty robust, and the results almost remain unchanged compared with Figure 4e.

**Figure 9:**
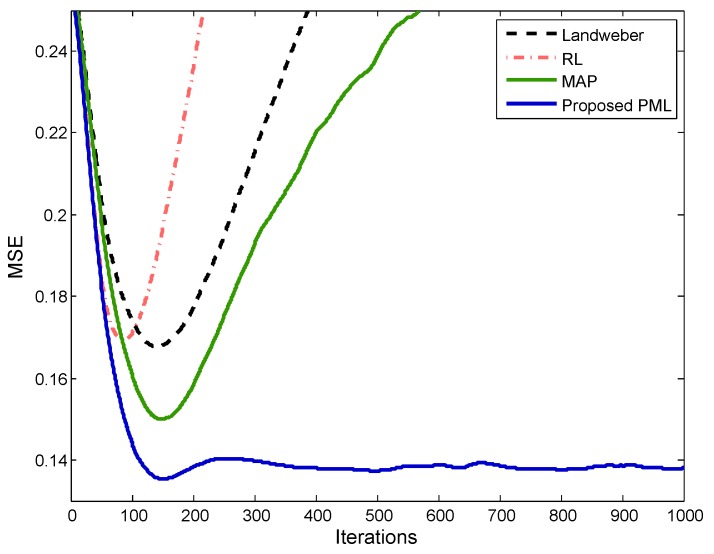
MSE curves comparison when SNR = 10 dB.

The MSE values of the four methods are illustrated in Figure 9, from which we can observe that the minimum MSEs of all the methods are smaller than that of Figure 7 due to the effect of the noise. In addition, the curvatures of the Landweber, RL and MAP are larger than that of Figure 7. It indicates that the super-resolution performance of these three methods are more sensitive to the iterations, and it is getting harder to find the optimal solution. On the contrary, the MSE curve of the proposed PML still presents itself to be stable, and the MMSE is much smaller than that of Landweber, RL and MAP. Clearly, the proposed method has a more outstanding performance in the low SNR cases.

### 4.2. Real Data Processing

**Figure 10:**
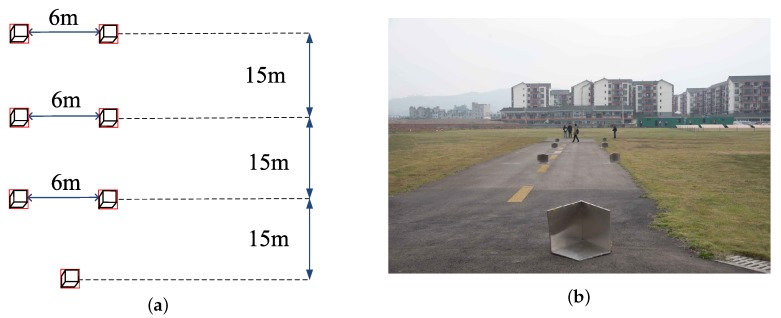
Experiment scenario (**a**) the diagram of the relative positions of the corner reflectors; (**b**) optical scenario.

In this subsection, the results of real data processing are provided to validate the effectiveness of the proposed method. The imaging scenario is composed of seven corner reflectors that are located on a clear runway. Figure 10a displays the relative positions of the corner reflectors and Figure 10b shows the optical picture of the scenario. A Ka band radar system is fixed at the bottom of a helicopter and used to irradiate the forward-looking region. The related experiment parameters are displayed in Table 3.

**Figure 11:**
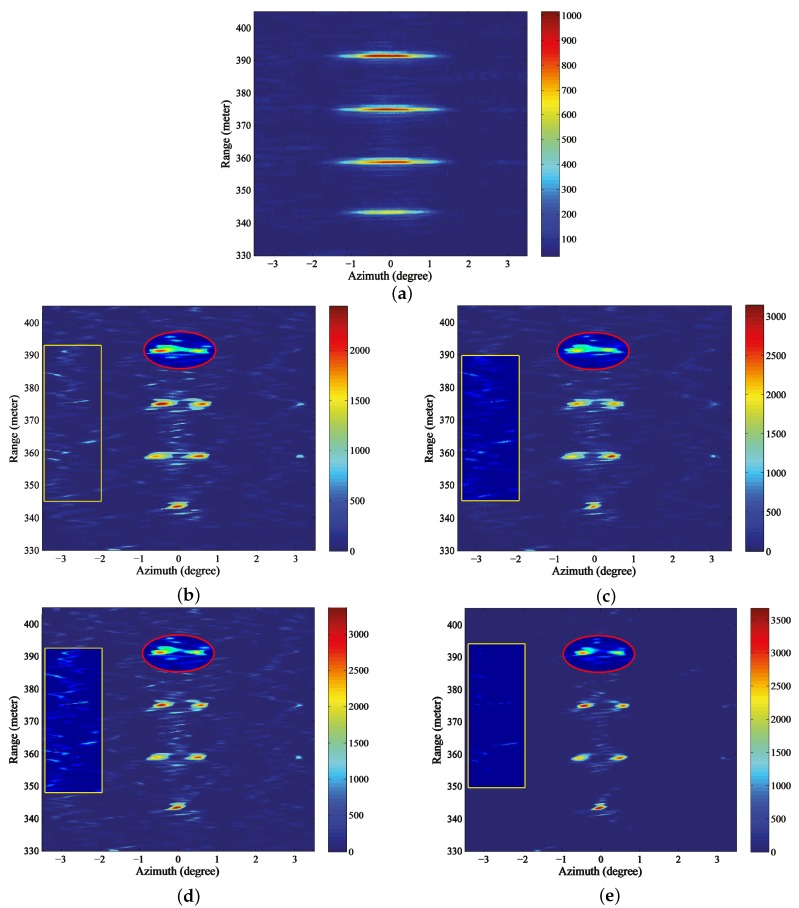
Angular super-resolution results for real beam data through automatically stopping the iterations (**a**) real beam echo after range compression and migration correction, results of (**b**) Landweber (**c**) Richardson-Lucy (RL) (**d**) maximum a posterior (MAP) (**e**) proposed penalized maximum likelihood (PML).

Figure 11a shows the echo of the Ka band radar after range compression and range migration correction. We can observe that the echo in Figure 11a mainly comes from the corner reflectors due to the relative small pitch angle. In addition, the echo of the corner reflectors are broadened and the adjacent reflectors that are located in the same range bin cannot be distinguished. Then, the Landweber, RL, MAP and the proposed PML are conducted in Figure 11a, respectively, and the iterative stopping criterion still follows the discrepancy principle. As to the estimation of the noise, since we concentrate on the internal noise produced by the radar system, we measured the output of the experiment radar in the quiet period (with no pulses transmitted) and took this measurement as the estimation of the noise standard deviation ρ. Then, the ISV of the discrepancy principle can be determined as $\sqrt{N}\rho$, where *N* is the length of one range cell. For most of the sweeps, this estimation is accurate enough to stop the iteration. However, on account of the noise being a statistical variant, there is a certain probability that this estimation cannot meet the requirement for iteration termination. Hence, for several sweeps, the iterative stopping criterion cannot cease the iterations properly for the traditional methods and we take one of these sweeps to demonstrate the advantage of the proposed method.

**Figure 12:**
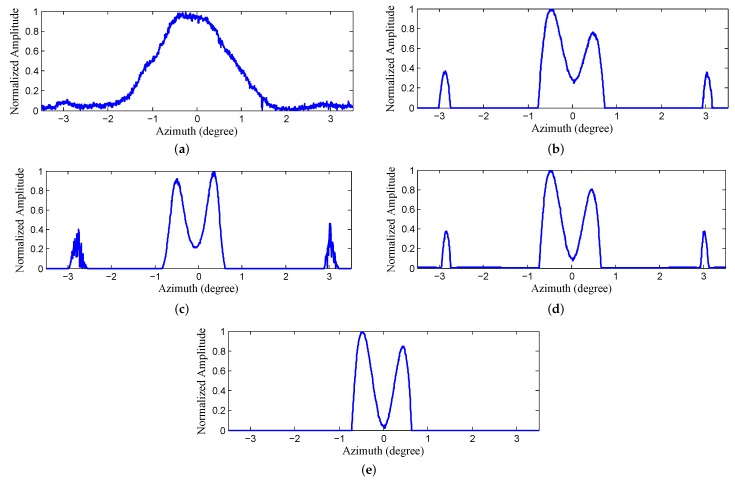
The profiles of the farthest corner reflectors in Figure 11 obtained by (**a**) real beam echo after range compression and migration correction (**b**) Landweber (**c**) Richardson-Lucy (RL) (**d**) maximum a posterior (MAP) (**e**) proposed penalized maximum likelihood (PML).

The results of Landweber, RL, MAP and PML are displayed in Figure 11b–e. It can be observed from Figure 11b,c that the closer corner reflectors are distinguished. However, the farthest two reflectors that are circled by the red ellipse are barely resolved due to the narrower angular distance. In addition, there are some spurious targets emerging in the background after being processed and a yellow rectangle is used to indicate the distinct false targets in the left. Figure 11d displays the results of MAP, from which we can observe that the farthest two reflectors are resolved and the resolution has been further improved. However, there are still many false targets. Figure 11e demonstrates the results of the proposed PML. It can be observed that not only are all the reflectors completely resolved, but there is also almost no false targets and the background is much clear. Figure 12a–e demonstrate the profiles of the the farthest two reflectors in Figure 11a–e, respectively. We can tell from the results of Landweber, RL and MAP that there are obvious false targets on both sides of the targets and the amplitudes of the false targets are pretty large because excessive iterations are conducted. However, the results of the proposed PML method are not influenced much by the iterations and the background is very clean.

**Figure 13:**
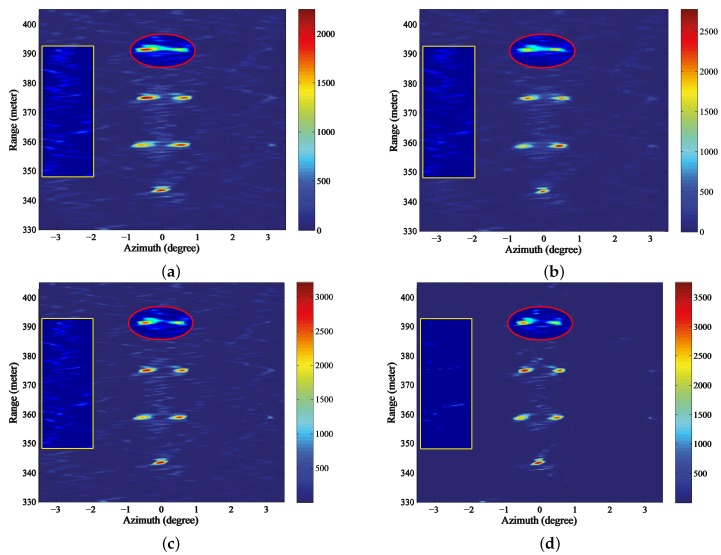
Angular super-resolution results for real beam data through manually adjusting the iteration numbers (**a**) Landweber; (**b**) Richardson-Lucy (RL) (**c**) maximum a posterior (MAP)(**d**) proposed penalized maximum likelihood (PML).

**Figure 14:**
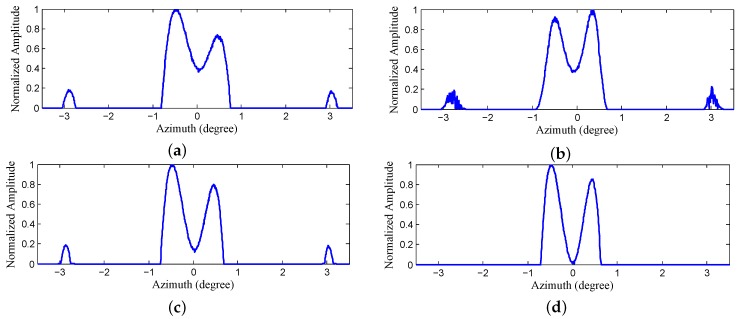
The profiles of the farthest corner reflectors in Figure 13 obtained by (**a**) Landweber (**b**) Richardson-Lucy (RL) (**c**) maximum a posterior (MAP) (**d**) proposed penalized maximum likelihood (PML) (**e**) proposed penalized maximum likelihood (PML).

Since there are many spurious structures that emerge when the iterations are automatically stopped, we manually adjust the iteration numbers to find the optimal super-resolution results by inspecting the performance in resolving the targets and suppressing the false targets. The final results are displayed in Figure 13. It can be observed from Figure 13a to Figure 13c that the performances of Landweber, RL and MAP have been improved compared with Figure 12a–c in terms of a good balance between resolving the targets and suppressing the spurious targets being achieved. As a contrast, the results of the proposed PML do not vary much compared with the results obtained by automatically stopping the iterations, which verifies the stability of the proposed method. The results of the profiles displayed in Figure 14 also verify the results in Figure 13. In addition, among the manually adjusted results, the results of the proposed PML still outperform the other three methods, and they not only possess a high resolution, but also can efficiently reduce the false targets.

## 5. Conclusions

In this paper, we have proposed a penalized maximum likelihood angular super-resolution method for scanning radar forward-looking imaging. This method can greatly enhance the noise resistance ability and efficiently conquer the noise amplification phenomenon of the statistical angular super-resolution method. Firstly, the acquisition process of raw data is analyzed and the likelihood is reduced through separately considering the noise in the I and Q channels to improve the accuracy of the noise modeling for the radar imaging system. Next, a joint square-Laplace penalty is formulated by making use of the outlier sensitivity property of square constraints as well as the sparse expression ability of Laplace distribution to resist the noise amplification and maintain the resolving ability. Finally, an accelerated iterative solution strategy is adopted to solve the obtained convex optimal problem to facilitate the engineering application of the proposed method. Experiments verify the effectiveness of the proposed method. Compared with traditional statistical super-resolution methods, the proposed method has a better super-resolution performance in reducing the spurious targets and enhancing the robustness to the noise amplification.

## Figures and Tables

**Table 1:** The flowchart of the accelerated iterative shrinkage/thresholding solution strategy.

**Initial Step**: Take $\sigma_{0}=s$, compute the first two iterative results $\sigma_{1}$ and $\sigma_{2}$ through the basic iteration formula Equation (36)
and the first two iterative vectors $g_{1}=\sigma_{1}-\sigma_{0}$ and $g_{2}=\sigma_{2}-\sigma_{1}$
**Repeat**
Compute the extrapolation step size
$\alpha_{k}=\frac{{\sum g_{k}\cdot g}_{k-1}}{{\sum g_{k-1}\cdot g}_{k-1}}\;\;\;\;\;\;\;\;\;\;\;\;\;\;\;\;\;\;\;0\;\;<\alpha_{k}<1$
Calculate the prediction point $y_{k+1}=\sigma_{k}+\alpha_{k}\left(\sigma_{k}-\sigma_{k-1}\right)$
Compute the next iterative result with the obtained predicted point through the basic iteration formula Equation (36)
$\sigma_{k+1}=\psi \left(y_{k+1}\right)$
Calculate the prediction point
$y_{k+1}=\sigma_{k}+\alpha_{k}\left(\sigma_{k}-\sigma_{k-2}\right)$
Compute the next iterative result $\sigma_{k+1}$ with the obtained predicted point $y_{k+1}$ through the basic iteration formula Equation (36), $\sigma_{k+1}=\psi \left(y_{k+1}\right)$
Update the iterative vector $g_{k+1}=\psi \left(y_{k+1}\right)-y_{k+1}$
**Until (convergence)**
**Output the iterative result $\sigma_{k+1}$**

**Table 2:** Simulation parameters.

Parameters	Values
Pulse repetition frequency (PRF)	4000 Hz
Antenna scanning velocity	$60^{\circ }$
Main-lobe beam width	$3^{\circ }$
Antenna scanning area	${\pm 10}^{\circ }$

**Table 3:** Experiment parameters.

Parameters	Values
Carrier frequency	30.75 GHz
Platform velocity	30 m/s
Pitch angle	$20^{\circ }$
Band width	200 MHz
Pulse repetition frequency (PRF)	4000 Hz
Pulse duration	1 μs
Antenna scanning velocity	$60^{\circ }$
Main-lobe beam width	$3.1^{\circ }$
